# An EEG-fMRI Study on the Termination of Generalized Spike-And-Wave Discharges in Absence Epilepsy

**DOI:** 10.1371/journal.pone.0130943

**Published:** 2015-07-08

**Authors:** Francesca Benuzzi, Daniela Ballotta, Laura Mirandola, Andrea Ruggieri, Anna Elisabetta Vaudano, Micaela Zucchelli, Elisabetta Ferrari, Paolo Frigio Nichelli, Stefano Meletti

**Affiliations:** 1 Department of Biomedical, Metabolic and Neural Sciences, University of Modena and Reggio Emilia, Modena, Italy; 2 Neurology Unit, NOCSAE Hospital-ASL, Modena, Italy; 3 Department of Psychology University of Bologna, Bologna, Italy; 4 IIT, Genova, Italy; University of Electronic Science and Technology of China, CHINA

## Abstract

**Introduction:**

Different studies have investigated by means of EEG-fMRI coregistration the brain networks related to generalized spike-and-wave discharges (GSWD) in patients with idiopathic generalized epilepsy (IGE). These studies revealed a widespread GSWD-related neural network that involves the thalamus and regions of the default mode network. In this study we investigated which brain regions are critically involved in the *termination* of absence seizures (AS) in a group of IGE patients.

**Methods:**

Eighteen patients (6 male; mean age 25 years) with AS were included in the EEG-fMRI study. Functional data were acquired at 3T with continuous simultaneous video-EEG recording. Event-related analysis was performed with SPM8 software, using the following regressors: (1) GSWD onset and duration; (2) GSWD offset. Data were analyzed at single-subject and at group level with a second level random effect analysis.

**Results:**

A mean of 17 events for patient was recorded (mean duration of 4.2 sec). Group-level analysis related to GSWD onset respect to rest confirmed previous findings revealing thalamic activation and a precuneus/posterior cingulate deactivation. At GSWD termination we observed a decrease in BOLD signal over the bilateral dorsolateral frontal cortex respect to the baseline (and respect to GSWD onset). The contrast GSWD offset versus onset showed a BOLD signal increase over the precuneus-posterior cingulate region bilaterally. Parametric correlations between electro-clinical variables and BOLD signal at GSWD offset did not reveal significant effects.

**Conclusion:**

The role of the decreased neural activity of lateral prefrontal cortex at GSWD termination deserve future investigations to ascertain if it has a role in promoting the discharge offset, as well as in the determination of the cognitive deficits often present in patients with AS. The increased BOLD signal at precuneal/posterior cingulate cortex might reflect the recovery of neural activity in regions that are “suspended” during spike and waves activity, as previously hypothesized.

## Introduction

Idiopathic Generalized Epilepsy (IGE) has been the target of several EEG-fMRI studies [1– 11]. These studies provided interesting and convergent insights into the neuronal networks involved in ‘generalized’ spike and wave discharges (GSWD) showing bilateral BOLD increases in the thalamus and decreases in the frontal and parietal cortical regions belonging to the default mode network (DMN) of the brain [12–14]. Studies on brain functional connectivity also showed that patients with absence seizures have an altered functional connectivity within the DMN and attentional networks. In particular, decreased functional connectivity has been observed in the precuneus/cingulate cortex, pre-frontal and parietal cortex, suggesting abnormal anatomo-functional architectural integration of brain networks in idiopathic generalized epilepsies [15–17]. Interestingly, functional connectivity showed a negative correlation with epilepsy duration [15, 16].

More recently, several authors have investigated the dynamics of BOLD signal before and during GSWD to define the precise role of the different cortical and sub-cortical areas in the generation of GSWD and their relative contribution to the impairment of consciousness [10,18–22]. In this respect, increments of the BOLD signal in cortical areas prior to thalamic involvement have been demonstrated. Results showed that cortical BOLD increments anticipate not only thalamic changes, but also the onset of GSWD recorded on the EEG.

Conversely, the BOLD signal changes related to seizure termination, in both focal and generalized epilepsies, have not been specifically investigated to date. This issue is relevant since the understanding of the mechanisms and dynamics involved in seizure termination can promote specific drug and/or neuro-modulatory therapies.

A few studies have focused on the mechanisms that promote seizures termination at molecular and cellular level [23]. Recently, a single-neuron recording study in human focal epilepsy reported that neuronal firing patterns change homogeneously at seizure offset suggesting that seizure termination is marked by an abrupt and homogeneous change in neuronal firing across different cortical patches [24]. These data support the theory that mechanisms acting at neural network level may have a relevant role in the determination of seizures offset. Only very recently, the cortico-thalamic circuits involved in animal models of absence epilepsy have been investigated [25]. It has been shown that the termination of spike-and-wave-discharges is a process, which is already initiated up to 1.5 seconds prior to discharge offset and involves both a decrease of cortico—thalamic coupling as well as intra-thalamic processes.

The evaluation of GSWD by means of EEG-fMRI could offer new insights in the investigation of the cortical and subcortical regions involved in GSWD termination. We evaluated these issues in a cohort of IGE patients with absence seizures (AS) focusing on the BOLD dynamics at discharge termination with respect to the neural network involved in spike and wave onset. We tested the hypothesis that, at AS offset, DMN areas, and particularly the precuneus, could present an increased neuronal activity mirrored by a relative increase in BOLD signal compared to the onset of the GSWD discharge. This would support the role of the precuneus/posterior cingulate cortex in the recovery of consciousness that clinically characterize the termination of the EEG discharge. We also, and more importantly, tested the hypothesis that at discharge offset some inhibitory mechanisms, acting at network level, can be the signature of GSWD termination. We hypothesize that a decreased, or negative, BOLD signal could be the fMRI marker of GSWD offset in a distributed network.

## Methods

Patients were recruited at the Epilepsy Center, Department of Biomedical, Metabolic and Neural Sciences of the University of Modena and Reggio Emilia, between April 2007 and December 2013. The patients’ inclusion criteria were the follows: 1) documented GSWD accompanied by impairment of consciousness during video-EEG monitoring; 2) routine EEG showing normal background; 3) normal developmental milestones; 4) normal structural imaging on anatomic MRI at 3 T.

AS were defined according to the International League Against Epilepsy [26].

The Human Ethic Committee of the University of Modena and Reggio Emilia, Italy granted approval for this project. Written informed consent was obtained from each subject participating in the study (or by his/her parents for subjects < 18yrs).

### Patients’ selection

Sixty-eight IGE patients underwent a video-EEG-fMRI recording in the study period. Thirty out of 68 fulfilled the inclusion criteria, and finally 21 were selected for the current EEG-fMRI analysis on the basis of the recording of GSWD discharges with similar duration and EEG morphology respect with electroclinical AS recorded during routine EEG.

Out of 21 patients, three subjects were discarded due to movement artifacts. Therefore the final study group consists of 18 patients (six male; mean age 25 years) whose clinical details are summarized in Table 1.

**Table 1:** Clinical data and details of EEG-fMRI.

Clinical data				EEG-fMRI co-registrations		
Pts	Age/ gender	Diagnosis	AEDs	Recording duration min.	N° events (GSWD)	Mean duration of GSWD sec.
pt1	24/F	JAE	LEV	30	45	3,24
pt2	33/F	JAE	VPA	20	15	3,61
pt3	47/M	JAE	VPA	10	21	3,71
pt4	48/F	CAE	VPA	20	14	4,03
pt5	26/M	Adult onset IGE	PB/TPM/ZNZ	40	12	4,99
pt6	37/F	JAE	LEV	20	19	3,91
pt7	15/F	JAE	VPA	20	16	3,18
pt8	23/F	Adult onset IGE	LEV	10	3	3,07
pt9	5/F	CAE	Naïve	10	5	5,20
pt10	20/F	JAE	Naïve	10	9	3,14
pt11	16/M	CAE	Naïve	20	5	7,29
pt12	9/F	CAE	Naïve	10	3	7,63
pt13	13/M	JAE	VPA	10	9	5,19
pt14	10/F	CAE	Naïve	10	6	5,07
pt15	44/F	Adult onset IGE	LEV	30	47	2,13
pt16	35/F	JAE	VPA	30	15	2,45
pt17	15/M	JME	LEV	10	8	3,40
pt18	28/M	JAE	VPA	20	41	3,74

JAE, juvenile absence epilepsy; CAE, childhood absence epilepsy; IGE, idiopathic generalized epilepsy; JME, juvenile myoclonic epilepsy; AEDs, antiepileptic drugs; VPA, valproate; LEV, levetiracetam; PB, phenobarbital; TPM, topiramate; ZNZ, zonisamide; GSWD, generalized spike-wave discharges

The syndromes classification was obtained by two expert epileptologists after a careful revision of clinical and electrophysiological data of each patient (S.M., A.E.V.). The epilepsy syndrome was defined based on the ILAE classification [26].

### Video-EEG acquisition

All scans were performed in the early afternoon. The patients were asked to fall sleep 2 hours later and wake-up 1 hour before than usual the night preceding the EEG-fMRI test. Sleep deprivation indeed is documented to determine vigilance level fluctuations that have an essential role in GSWD activation in wakefulness and even in superficial sleep [27]. Hyperventilation was not used to induce GSWD. Scalp EEG was recorded by means of a 32 channels MRI-compatible EEG recording system (Micromed, Mogliano Veneto, Italy). Electrodes were placed according to conventional 10–20 locations. FCz was the reference. ECG was recorded from 2 chest electrodes. Electrode impedance was kept below 10 kOhms. Prior to in-magnet EEG recording, 10 min of out-of magnet EEG data were collected in a room adjacent to the scanner. Foam pads were used to help secure the EEG leads, minimize motion, and improve patient comfort. Data were transmitted via an optic fiber cable from the high-input impedance amplifier (1.024 Hz sampling rate) to a computer located outside the scanner room. To avoid saturation, the EEG amplifier had a resolution of 22 bits with a range of ±25.6 mV. An anti-aliasing hardware band-pass filter was applied with a bandwidth between 0.15–269.5 Hz.

Patients’ behavior was constantly observed and recorded by means of a small camcorder positioned on the head coil inside the scanner pointing to the patient’s face to obtain a split-screen video-EEG documentation during the fMRI recording [28].

### fMRI acquisition

Functional data were acquired using a Philips Intera system at 3T and a gradient-echo echo-planar sequence from 30 axial contiguous slices (TR = 3000 ms; in-plane matrix = 64x64; voxel size: 4x4x4 mm) over three 10-min sessions per patient with continuous simultaneous video-EEG recording. A high-resolution T1-weighted anatomical image was acquired for each patient to allow anatomical localization. The volume consisted of 170 sagittal slices (TR = 9.9 ms; TE = 4.6 ms; in plane matrix = 256x256; voxel size = 1x1x1 mm).

### EEG and fMRI data processing

The correction of the gradient artifact was performed offline by means of the Brain Quick System Plus software (Micromed, Mogliano Veneto, Italy)[29]. Following, the EEG data were exported in the.edf format and reviewed and analyzed by means of the BrainVision Analyzer 2.0 software (Brain Products, Munich, Germany). A bandpass filter between 1 and 70 Hz was applied to the continuous recording and channels showing high impedance or electrode displacement artifacts were interpolated through a cubic spline. Pulse related artefacts were removed offline from the EEG trace recorded during scanning using the EEG processing package of Brain Analyzer (Brain Products, Munich, Germany)[30]. The pre-processed EEG data were then submitted to EEG Independent Component Analysis (ICA) for a further recognition of the components carrying residual physiological artefacts (blinks, saccades and heartbeat) and the GSWD activity (S1 Fig)[31,32]. To optimize artifactual activities removal, blinks and saccades were marked on channel Fp1; R-peaks due to cardiac artifact were also marked for subsequent artifact removal when present. ICA was applied at the single-subject level in order to separate the generators of EEG activities and maximizing the statistical independence among them. For each participant, the 30 EEG channels signal was decomposed in 30 components (between F0 to F29). Each component resulting from ICA separation is characterized by a time course, describing the morphology of the component over time, and by a specific topography (i.e. an array containing the weights the specific component has on each channel). To determine the components carrying the most typical EEG artifacts (blink, saccades and cardiac) we separately segmented and averaged each component on the basis of each artifact marker. Subsequently, the amount of variance each component contributed to the averaged evoked activity was computed. Finally, the components that amounted more than the 50% of global variance were marked as artifactual components [28,33].

To detect GSWD, two expert epileptologists (S.M., A.E.V.) reviewed both the standard EEG recordings and the relative individual components of the ICA-processed recordings. The independent components that on visual inspection showed GSWD activity were marked as Components Of Interest (COIs). Following, absence seizures onset and offset were marked on the COIs showing the maximum amplitude spike-and-wave complexes. S1 Fig shows the results derived from ICA in a representative patient. The number of COIs varied from 2 to 5 for each patient.

The Matlab 7.11 and SPM8 (Wellcome Department of Imaging Neuroscience, London, UK) software were used for fMRI data analysis. For each patient, functional volumes were slice time corrected, realigned to the first volume acquired, normalized to the MNI (Montreal Neurologic Institute) template implemented in SPM8, and smoothed with a 12x12x12 mm FWHM Gaussian kernel. Data analysis was performed by means of the SPM8 general linear model using regressors of interest convolved with the standard hemodynamic response function (HRF).

The following two types of events were implemented as regressors in the single patient-first level analysis:

- **AS**: represented as variable duration events modeled by onset and duration; the onset was marked as the first spike component of the GSWD;
- **GSWD offset**: the onset of this regressor was marked by the last slow-wave component of each discharge; durations were considered to be equal to zero.

Rest was considered the period in which no abnormalities were detected on the EEG signal; it was modeled implicitly as baseline condition. Movement artifacts detected by the video-EEG recordings (head movement, lip, jaw movements, swallowing, blinking) were included as events of no interest. The movement parameters estimated during the realignment step were also included as nuisance regressors in the first level analysis.

For each patient, specific contrasts were settled up to test the following effects:

1. The BOLD effect of spike-and-waves with respect to the resting EEG background.
2. The BOLD changes at spike-and-waves termination with respect to the resting EEG background (GSWD offset *versus* rest) and respect with the onset of the discharge (GSWD offset *versus* GSWD onset).

Single patient contrast images were then submitted to a second level random effect analysis with separates one sample T-tests including age as covariate. A double statistical threshold (intensity and spatial extent) was adopted: only clusters with a voxel-wise intensity threshold of p < 0.001 (uncorrected) and a spatial extent threshold of p < 0.05 (uncorrected) were considered to be significant [34].

Coordinates in Talairach space [35] were obtained by applying the Matthew Brett correction (mni2tal: http://www.mrc-cbu.cam.ac.uk/Imaging/mnispace.html) to the SPM—MNI coordinates.

### ROI analysis

We performed an event-related time-course analysis on GSWD using the regions of interest (ROIs) identified on the group activation/deactivation maps. In particular, ROIs were centered on the activated/deactivated areas in the following contrast of the random effect analysis: GSWD-offset *versus* rest (deactivation) for the left frontal cluster (-34–8 46); GSWD-offset *versus* GSWD-onset for the precuneus (-10–52 14); GSWD-onset *versus* rest for the thalamus (6–4 2). ROI’s shape and size corresponded to the functional cluster transformed into binary maps. The BOLD signal time course was extracted from each ROI for each subject [by means of MarsBaR (http://marsbar.sourceforge.net/)] and used to calculate an average event-related response. The response was estimated from 6 seconds prior to 21 seconds following the event (the GSWD onset).

### Correlations with clinical variables

To test for a linear or higher order polynomial relation between BOLD signal changes relative to GSWD termination and clinical and neurophysiological measures we performed separate correlation analysis. Clinical and neurophysiological measures obtained during the scanning sessions included: age at epilepsy onset; duration of disease; GSWD duration; number of GSWD recorded; amplitude (μV) of the last slow-wave of the GSWD complex [calculated from the first negative peak (N1) to the positive peak (P1) of the slow-wave]; duration (ms) of the last slow-wave [calculated from the N1 to second negative peak (N2) of the slow wave].

## Results

A total of 233 events (mean of 17 events per patient) were acquired, from a minimum of three AS in subject #8 and #12, to a maximum of 47 events in subject #15. The duration of GSWD ranged from 2 seconds to 40 seconds, with a mean duration across subjects of 4.2 seconds (see Table 1). No patient had a period of sleep during EEG-fMRI, defined on EEG absence of vertex waves, sleep spindles and K-complexes.

As expected, the second-level group effect of AS compared with the resting interictal baseline replicated previous findings. Indeed we observed an increased BOLD signal in the thalamus. Significant clusters of activations were also found in the occipital cortex (cuneus and middle occipital gyrus; Brodmann Area (BA) 18 and 19) as well as in the superior frontal gyrus (BA 6; see S2 Fig and S1 Table). Regions of decreased signal comprised the precuneus/posterior cingulate region, and two symmetrical clusters located in the temporal/parietal cortex of both hemispheres (superior and middle temporal gyrus, supramarginal, angular, inferior parietal gyrus, BA 39, 40; S1 Table).

### GSWD offset effect

**Fig 1:**
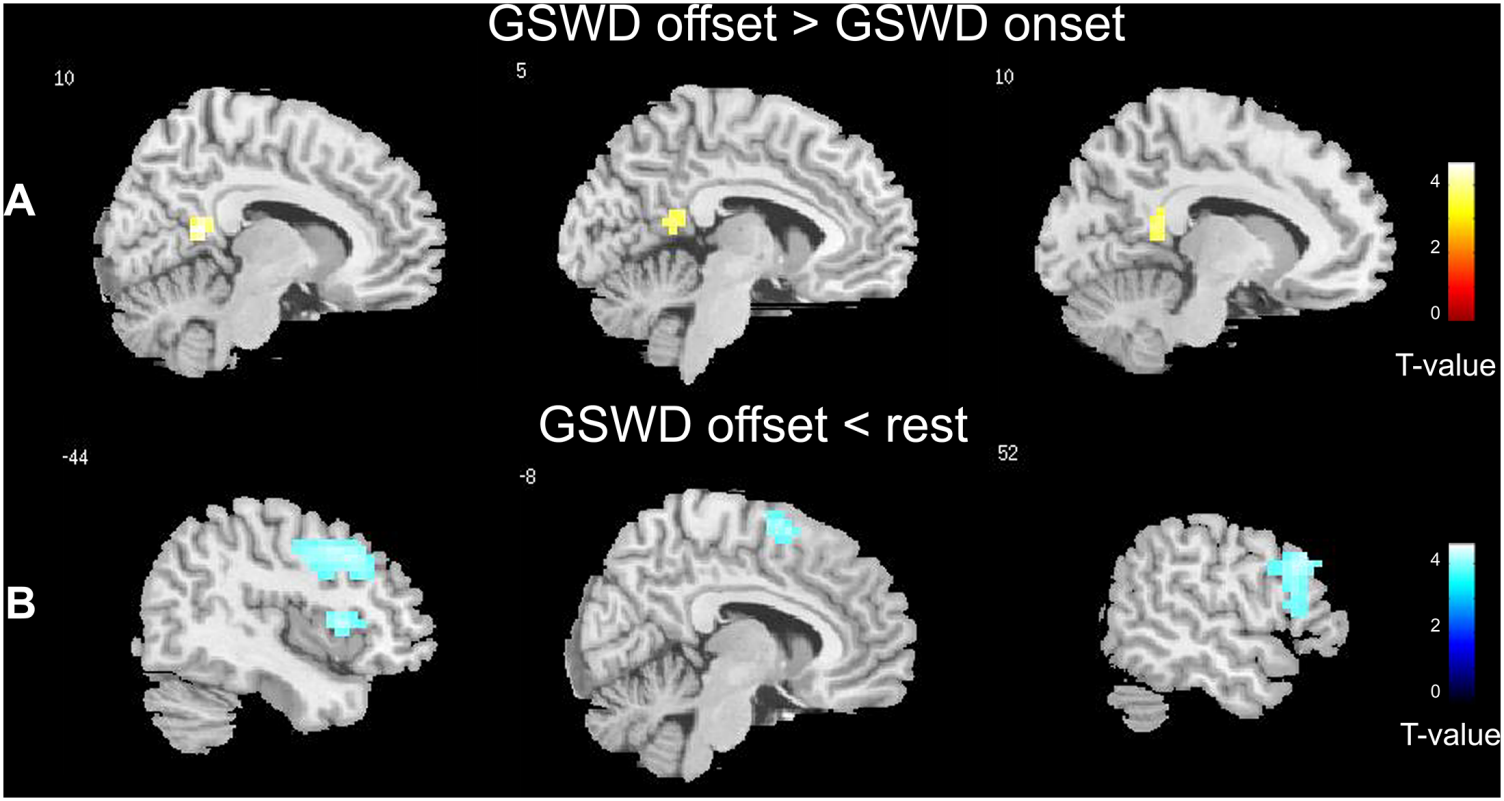
Brain regions showing BOLD signal changes at absence seizures termination. **A**. Areas of increased signal for the condition GSWD offset versus GSWD onset. **B**. Areas of decreased signal for the condition GSWD offset versus rest. Maps are superimposed on the template image MRICro (http://www.mccauslandcenter.sc.edu/mricro/index.html) and are thresholded at p ≤ 0.001 uncorrected for multiple comparisons, with a cluster extent threshold (k) of 10 voxels (p ≤ 0.05). Color bar represents T-values.

**Fig 2:**
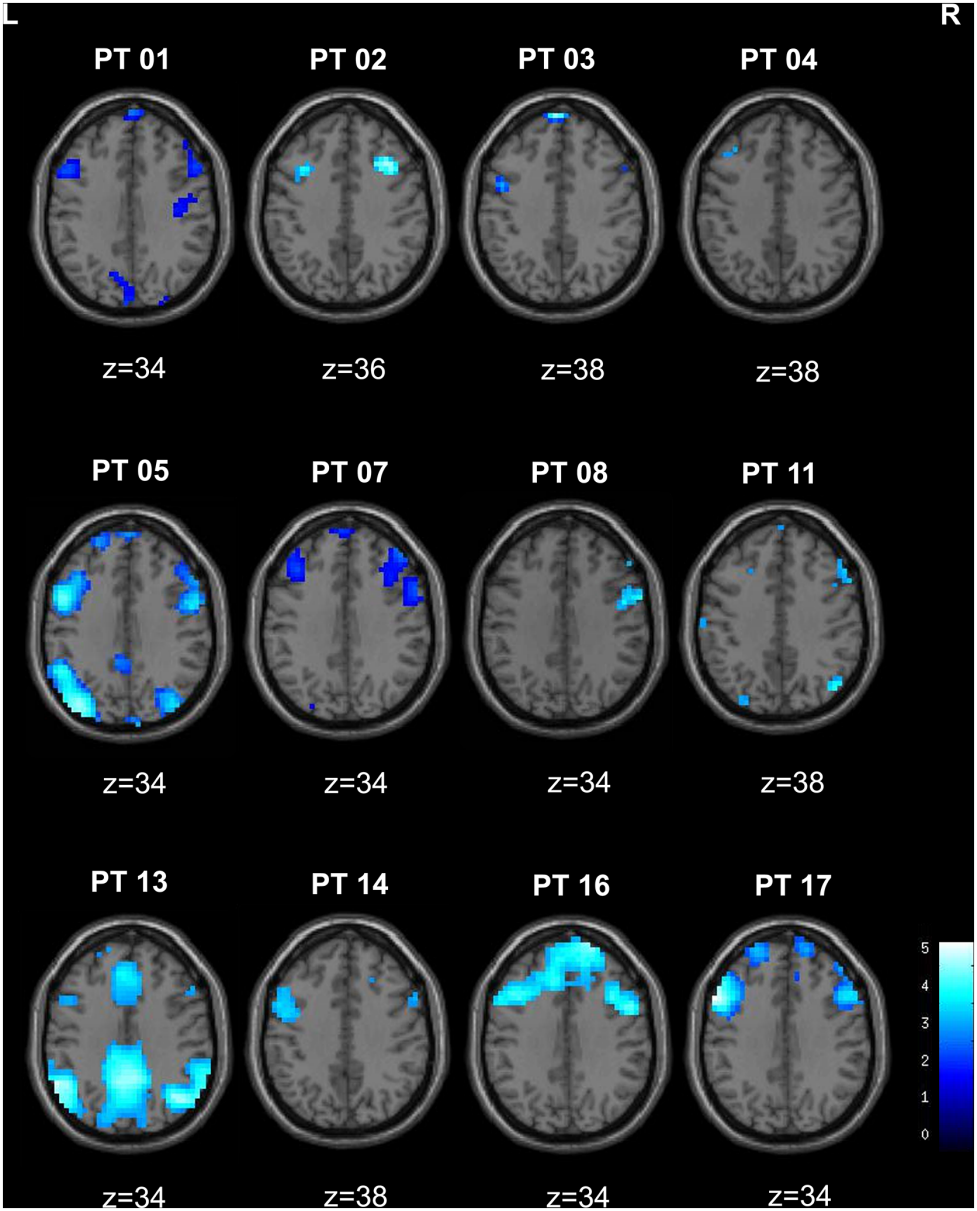
The Dorsolateral prefrontal cortex at absence seizure termination. Decrease BOLD signal at GSWD termination was observed in 12 patients. Axial sections displaying negative BOLD signal changes in single patients for the contrast GSWD offset versus rest; numbers represent MNI coordinates. Maps are superimposed on the MRICro (http://www.mccauslandcenter.sc.edu/mricro/index.html) template and are thresholded at p ≤ 0.001 uncorrected for multiple comparisons, with a cluster extent threshold (k) of 10 voxels (p ≤ 0.05). Color bar represents T-values.

No significant BOLD increase was observed at spike-and-waves termination compared with the resting normal EEG background (*GSWD* offset versus rest). BOLD signal increases at spike-and-waves termination were found in the precuneus/posterior cingulate region comparing GSWD offset with respect to GSWD onset (Table 2; Fig 1A). On the contrary, frontal regions showed a decrease in BOLD signal either comparing GSWD offset with respect to the resting baseline, as well as with respect to the GSWD onset: clusters of decreased signal occurred in the dorso-lateral frontal cortex of both hemisphere and particularly in the left precentral, middle and inferior frontal gyrus (BA 4, 9) and in the right middle and inferior frontal gyrus (BA 9, 44, 8; Table 3; Fig 1B). A decrease of BOLD signal at GSWD offset involving the frontal cortex was consistently observed also at single-subject level. Indeed 12 out of 18 patients showed a deactivation of the dorsolateral prefrontal cortex at discharge offset at a threshold of p < 0.001 uncorrected (Fig 2).

**Table 2:** Region of increased signal for the contrast: GSWD offset versus GSWD onset (cluster level: p < 0.001 not corrected, voxel level: p < 0.05).

*Anatomical region*	*Spatial Coordinates*								
				*MNI*			*Tal*		
	*Side*	*k*	*Z*	*x*	*y*	*z*	*x*	*y*	*z*
Posterior cingulate, Precuneus	R	74	4,13	-10	-52	14	-10	-50	15
	L	70	3,85	10	-48	14	10	-46	15

R = Right; L = Left

**Table 3:** Region of significant difference for the contrast GSWD offset versus rest (cluster level: p < 0.001 not corrected, voxel level: p < 0.05).

*Anatomical region*	*Spatial Coordinates*								
	*Side*	*k*	*Z*	*MNI*			*Tal*		
				*x*	*y*	*z*	*x*	*y*	*z*
**Increasing BOLD**									
No significant voxel									
**Decreasing BOLD**									
Precentral. Middle frontal gyrus (BA 4, 9)	L	320	3,67	-34	-8	46	-34	-6	43
			3,65	-42	4	38	-42	6	35
			3,50	-6	4	62	-6	7	57
Middle and inferior frontal gyrus (BA 9, 44, 8)	R	101	3,52	54	20	34	53	21	30
			3,32	38	8	30	38	9	27
			3,22	34	12	42	34	14	38
Inferior frontal gyrus (BA 45)	L	30	3,46	-50	16	6	-50	16	5
Superior frontal gyrus (BA 8)	R	27	3,22	18	36	54	18	37	48

L = Left; R = Right; BA: Brodmann Area

### ROI analysis

**Fig 3:**
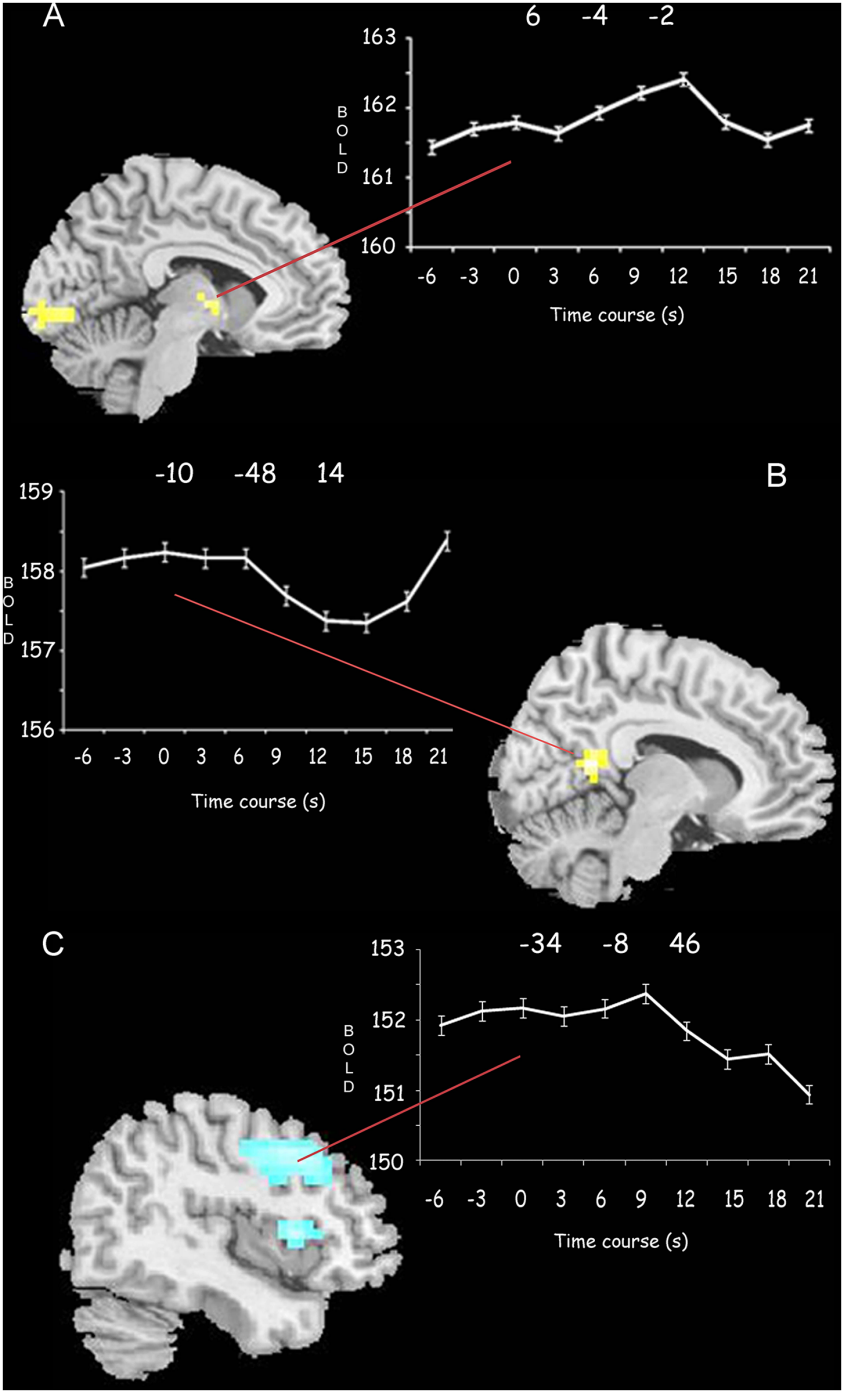
ROIs signal time courses. **A**. Shows the mean BOLD time-course of the thalamus (contrast GSWD onset respect to rest: MNI coordinates for ROI: 6–4 2). **B**. BOLD time course of the PCC (contrast GSWD offset versus GSWD onset coordinates for this ROI was: -10–52 14). **C**. Shows the BOLD time-course of the frontal ROI (contrast GSWD offset versus rest; MNI coordinates: -34–8 46). In each graph the time ‘0’ represents the onset of the spike and wave discharge.

Raw signal time course confirmed that the thalamus increased its activity at the onset of the GSWD and decreased slowly (Fig 3). On the contrary, frontal areas showed a small BOLD signal increase at GSWD onset followed by a decrease after three seconds post-onset (Fig 3). Conversely, the BOLD signal of the precuneus decreased at GSWD onset and started to increase approximately after five seconds.

### Correlations with electro-clinical variables

No correlations were observed between BOLD signal at GSWD offset in relation to age at epilepsy onset; duration of disease; number of GSWDs recorded; amplitude and duration of the last slow-wave.

## Discussion

In this study we specifically investigated BOLD dynamics related to discharge termination marking, for each single EEG event, the final slow-wave of the discharge. In this way we were able to inform the single-subject fMRI design (and consequently the group analysis) to reveal more precisely the neural dynamics time-related to the offset of the epileptic discharge. By means of this approach we demonstrated that GSWD offset was associated with increased BOLD signal in the PPC, and with decreased signal in the dorsolateral prefrontal cortex (DLPFC) of both hemispheres. These results were consistent across patients and thus represent the hemodynamic correlate of the neural mechanisms underpinning discharge termination. In particular, our results emphasize the importance of DLPFC in the cortical networks that are related to “generalized” spike-and-wave complexes and to absence seizures, suggesting that the frontal lobe has an important role, or it is critically involved, in the neural dynamics related to the termination of AS. Notably, we propose that the negative BOLD response (NBR) in the DLPFC can represent a core feature of GSDW offset in the same way that thalamic activations and DMN deactivations represent the core feature of GSWD generation in different patients’ populations belonging to genetic generalize epilepsies (CAE, JAE, JME) [1– 11]. Interestingly, the contribution of frontal lobe networks in AS was recently highlighted in an EEG-fMRI study and it has been proposed that the timing of the BOLD changes in frontal cortical areas might identify sub-groups of patients [36]. Notably the DLPFC regions showing a NBR at absence seizure termination in this study involved frontal lobe areas that have been demonstrated to show altered functional as well as structural connectivity in IGE patients’ populations [15–17,37,38]. Future studies should address if these abnormalities in frontal lobe connectivity can be related, or can contribute, also to the mechanisms associated to AS termination.

Heterogeneity across patients’ electro-clinical features and age was present in the selected population. However, with respect to the aim of the study, that was to investigate the brain networks underlining the GSWD phenomenon *per se*, we believe that the heterogeneity of the population in term of IGE sub-syndromes does not undermine our results. Indeed, absence seizures and the relative EEG pattern are common features of different IGE sub-syndromes, sharing both clinical and EEG aspects. In this view, previous published data showed a uniform GSWD-related BOLD pattern across different IGE sub-syndromes, with different disease duration, age, and treatments, thus supporting our approach [2–11, 39]. These findings lend support to the notion that the pathological EEG discharge *per se*, rather than the clinical expression of the syndrome, could be the key determinant of the hemodynamic variations in a given brain network. In other words, EEG-correlated fMRI can be thought of producing ‘images of the network involved during the EEG feature’. Beyond these consideration it must also be noted that the results obtained in our patients’ population concerning the BOLD correlates of GSWD onset were totally concordant with previous findings: increased thalamic BOLD signal, as well as decrease of BOLD signal in the precuneus/posterior cingulate region (PPC) and in other regions belonging to the DMN [4,7,10]. This means that the EEG and related BOLD dynamics of the investigated patients are representative of the idiopathic ‘generalized’ epilepsies. Finally, the potential variability due to the patients’ age was addressed including the age as covariate in second-level fMRI analyses.

### Meanings of the observed BOLD changes at AS terminations

We observed that AS termination corresponds to PPC increased BOLD signal with respect to AS onset. The PCC seems to play an important role within the resting state network. It shows the highest level of metabolic activity at rest [13], it is considered the principal hub of the DMN [12], and the neural substrate for the maintenance of the conscious state [40]. According to this view, the increased BOLD signal at precuneal/posterior cingulate cortex at AS termination might reflect the recovery of neural activity in regions that are “suspended” during spike and waves activity. As far as the observation that AS termination was associated with a bilateral decrease of BOLD signal in the DLPFC this result can be interpreted according to different hypotheses. While, in general, the positive BOLD response (PBR) has been related to increased neuronal and synaptic activity [41], the significance of the NBR is less well understood. Theoretically, the NBR could represent a purely hemodynamic effect due to a reduction of the cerebral blood flow (CBF) in the deactivated areas secondary to a CBF increase in the activated regions, or the undershoot of an earlier positive hemodynamic change occurring before, or during, the GSWD [42,43]. Both these explanations seems unable to account for the negative BOLD in the DLPFC at AS offset. Indeed, the only BOLD signal increase at GSWD termination was observed in the PPC region that is subserved by a different cerebrovascular territory respect with the DLPFC. Moreover the DLPFC regions didn’t show a consistent positive BOLD response during AS, as revealed by the second-level random effect analysis (and also in the ROI event-related time-course analysis). Finally, the NBR of the DLPFC was present not only in comparison with AS onset, but also relative to the resting brain state. We believe that the NBR reflect a decrease in neuronal activity under its basal level, with corresponding decrease of CBF and oxygen consumption in DLPFC regions [44–48]. As we considered the last slow-wave of the GSWD complex to mark out the end of absence seizures, we hypothesize that the decreased signal in the DLPFC reflects the neural events underlying slow-wave generation. Recently, a relationship between deactivations and the presence of slow-waves in the interictal epileptiform discharges has been demonstrated, suggesting that NBR reflects neuronal inhibition probably mediated by GABA inhibitory interneurons activity involved in the slow-wave generation that follows the spike activity [49]. Experimental studies have shown that slow waves are caused by a robust hyperpolarization of the membrane of the pyramidal cells of the third and fifth cortical layers [50]. There are different opinions about the fact that the hyperpolarization associated with slow waves is a process that requires energy or not. On one hand it has been demonstrated with multiple intracellular recordings that virtually all neurons (local inhibitory interneurons included) are silent during this phase [51], suggesting that the neuron membrane is in a ‘‘refractory mode”. This hypothesis would imply that for this process no energy is required. On the other hand, it has been shown that this hyperpolarization is due to GABA inhibitory interneurons. Nevertheless, it has been shown that due to their fast connections and the position of their synapses along the soma of the neurons, inhibitory interneurons are very efficient in inhibiting a high number of neurons despite a low consumption of energy [52,53]. Therefore, even if the hyperpolarization is an active process, it could cause a decrease in neuronal activity with respect to the baseline.

### Study limitations

Our study has several limitations that must be taken into consideration.

First, our approach presents some methodological limitations since we used two non-orthogonal regressors to model GSWD onset and offset as events of interest. Indeed, we used two event of interest that are intrinsically related, few seconds apart, and of which the second depends from the first. Considering the HRF model and the slowness of the fMRI signal some criticality exists. However, we believe that in our experimental conditions, with a high number of events (total > 200; > 15 per subjects), and with a mean events’ duration of about four seconds the fMRI model can really detect the BOLD dynamics at GSWD offset. Importantly, with respect to the fMRI signal, it is not its slowness that matters, but rather the sampling rate. In a situation in which there is a good enough signal-noise ratio (a large enough sample), it is possible to discriminate two (consistent) events a fraction of a second apart.

Second, we could not obtain behavioral measures of consciousness/attention during the fMRI recordings. However, we recruited for this study (see methods section) only subjects with confirmed AS on video-EEG and with GSWD during fMRI recordings of a length similar to the AS recorder in the EEG lab. Therefore, we are confident that the events analyzed were associated, at least in the majority of the cases, with some impairment in attention/consciousness.

Third, we have not investigated the potential role drugs on AS termination. Indeed AEDs could exert their effect in the way GSWD terminates. In our patients’ group, five subjects were drug-naïve, seven were in monotherapy with valproate, five in monotherapy with levetiracetam, and one patient was in poly-therapy. Therefore, it was not possible to assess the effects of AED in direct group comparisons given the low sample size for second-level fMRI analyses. However, it should be noted that we observed BOLD decreases at discharge offset in the frontal cortex at single-subject level independently from treatment choices: in two drug naïve patients, in six patients in valproate treatment, in three patients treated with levetiracetam, and also in the patient in polytherapy. Of course these findings do not mean that AEDs have no effects on GSWD termination and this important topic needs to be addressed in future studies.

Finally, we were not able to find out any correlations between the negative BOLD response in the frontal cortex and several clinical/neurophysiologic variables. These negative results should be considered with caution due to the low sample size of our population and we believe they deserve future reconsideration in multicentric studies.

## Conclusions

To our knowledge this is the first study directly investigating the brain regions hemodynamically involved during GSWDs termination. Our findings support a crucial role of the PPC cortex in the alteration of conscious awareness that is disrupted during AS and is followed by a BOLD signal recovery time-locked to AS termination. Moreover, we showed that AS termination is associated to a bilateral negative BOLD response over the DLPFC. More data are need to understand the role of this decrease of BOLD signal and its eventual role in the brain mechanism involved in seizure termination. However, our results underscore the importance of the frontal lobe networks in the neural dynamics involved in AS.

## Supporting Information

S1 Fig Pre-processing steps of EEG data. All the displayed images refer to the same EEG page across different analyses in one representative patient. The EEG is displayed in bipolar montage. **A**. Left image: raw data EEG recording during fMRI acquisition (Micromed recording system). Note the presence of the gradient artifact, which obscures the EEG signal (Repetition Time-TR = 3sec). Right Image: EEG recorded during fMRI, after gradient artefact removal. The gradient artefact has been removed by means of the Brain Quick System Plus software (Micromed, Mogliano Veneto, Italy). **B**. Left Image: EEG recording during fMRI as imported in BrainVision Analyzer 2.0 software (Brain Products, Munich, Germany), after filtering (high pass filter: 1 Hz; low pass filter: 70 Hz). Note the presence of pulse-related artifact diffuse over all derivations. Right Image: EEG recording during fMRI after pulse artefact removal using a commercial EEG processing package (Brain Analyzer, Brain Products, Munich, Germany). **C**. Left Image: ICA analysis result. 30 Component were extracted (from F0 to F29). Within them the component of interests (COIs) are represented by the components F01, F02, F06, F08, F12 (underlined in blue). The COIs have been selected based on visual inspection by two expert epileptologists (S.M., A.E.V.). Right Image: COIs with the marked GSWD onset and offset.
(TIF)

S2 Fig Brain regions showing BOLD signal changes at absence seizures initiation. **A**. Areas of increased and **B**. decreased signal for the condition GSWD versus rest (p < 0.001 uncorrected; k ≥ 10 voxels). Blobs are superimposed on the template image of MRICro (http://www.mccauslandcenter.sc.edu/mricro/index.html). Color bar represents T-values.
(TIFF)

S1 Table Regions of significant difference in BOLD signal for the contrast: GSWD versus Rest (p < 0.001 uncorrected, k ≥ 10 voxels). (DOCX)
