# Supplementary figures and images for: An EEG-fMRI Study on the Termination of Generalized Spike-And-Wave Discharges in Absence Epilepsy
Source: PLoS One. 2015 Jul 8;10(7):e0130943. doi: 10.1371/journal.pone.0130943 (PMC4496065; doi:10.1371/journal.pone.0130943)

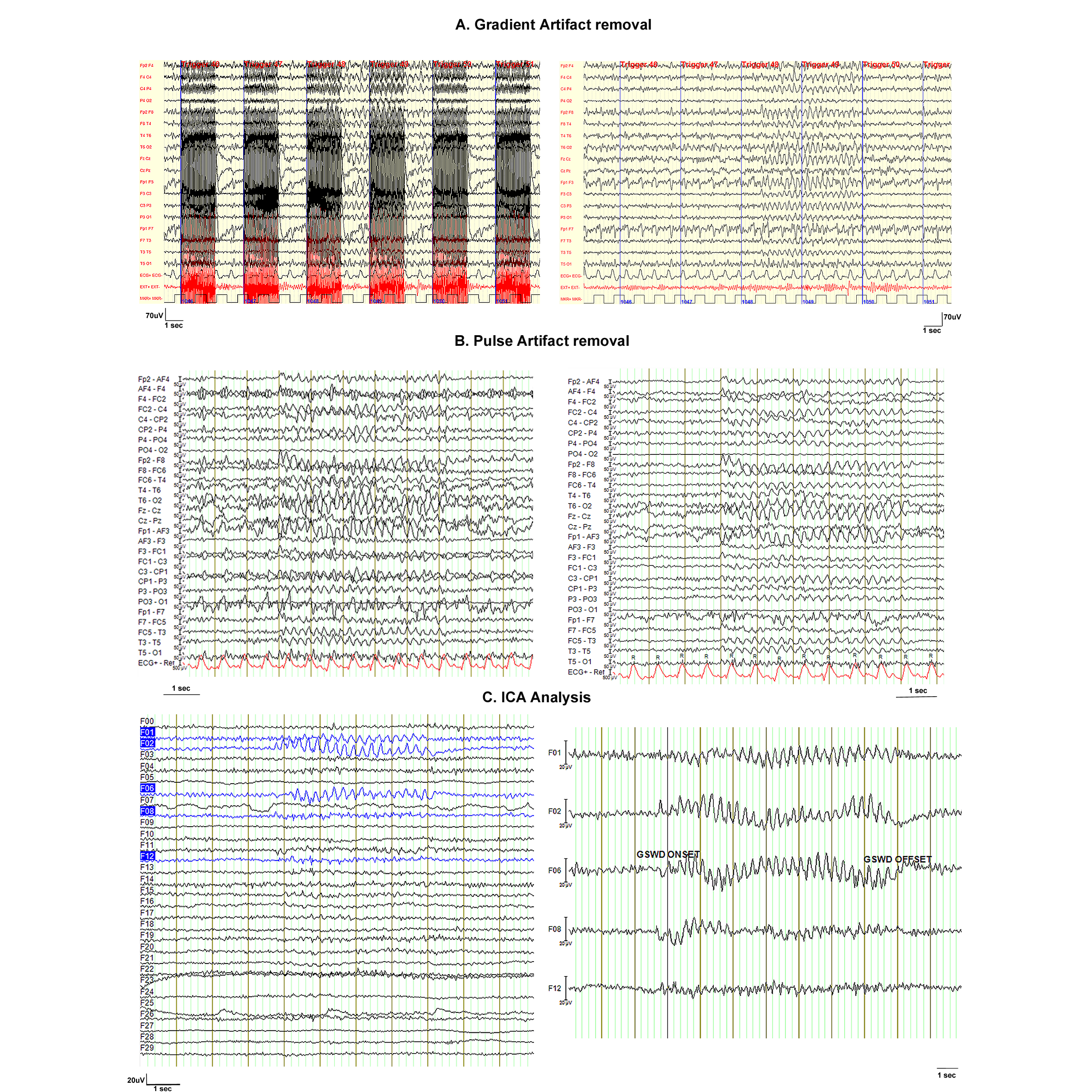

Supplement: S1 Fig — All the displayed images refer to the same EEG page across different analyses in one representative patient. The EEG is displayed in bipolar montage. A. Left image: raw data EEG recording during fMRI acquisition (Micromed recording system). Note the presence of the gradient artifact, which obscures the EEG signal (Repetition Time-TR = 3sec). Right Image: EEG recorded during fMRI, after gradient artefact removal. The gradient artefact has been removed by means of the Brain Quick System Plus software (Micromed, Mogliano Veneto, Italy). B. Left Image: EEG recording during fMRI as imported in BrainVision Analyzer 2.0 software (Brain Products, Munich, Germany), after filtering (high pass filter: 1 Hz; low pass filter: 70 Hz). Note the presence of pulse-related artifact diffuse over all derivations. Right Image: EEG recording during fMRI after pulse artefact removal using a commercial EEG processing package (Brain Analyzer, Brain Products, Munich, Germany). C. Left Image: ICA analysis result. 30 Component were extracted (from F0 to F29). Within them the component of interests (COIs) are represented by the components F01, F02, F06, F08, F12 (underlined in blue). The COIs have been selected based on visual inspection by two expert epileptologists (S.M., A.E.V.). Right Image: COIs with the marked GSWD onset and offset. (TIF) [file pone.0130943.s001.tif]

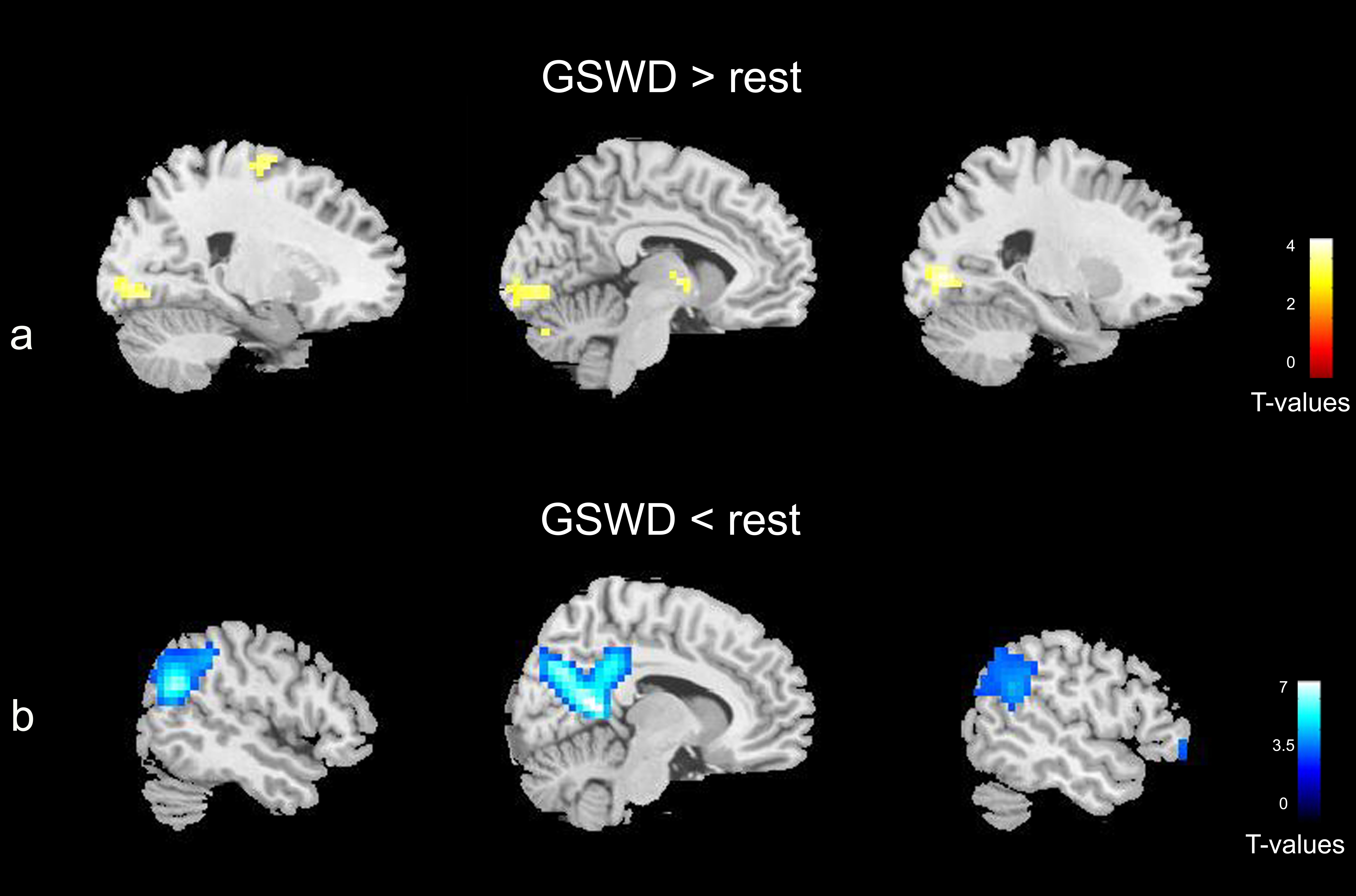

Supplement: S2 Fig — A. Areas of increased and B. decreased signal for the condition GSWD versus rest (p < 0.001 uncorrected; k ≥ 10 voxels). Blobs are superimposed on the template image of MRICro (http://www.mccauslandcenter.sc.edu/mricro/index.html). Color bar represents T-values. (TIFF) [file pone.0130943.s002.tiff]
