# Supplementary material for: An EEG-fMRI Study on the Termination of Generalized Spike-And-Wave Discharges in Absence Epilepsy
Source: PLoS One. 2015 Jul 8;10(7):e0130943. doi: 10.1371/journal.pone.0130943 (PMC4496065; doi:10.1371/journal.pone.0130943)
Supplement: S1 Table — (DOCX) [file pone.0130943.s003.docx]

**Supplementary Table. Regions of significant difference in BOLD signal for the contrast: GSWD versus Rest (p < 0.001 uncorrected, k > 10 voxels).**

| ***Anatomical region*** |  |  |  | ***Spatial Coordinates*** | | | | | |
| --- | --- | --- | --- | --- | --- | --- | --- | --- | --- |
|  |  |  |  | ***MNI*** | | | ***Tal*** | | |
|  | ***Side*** | ***k*** | ***Z*** | ***x*** | ***y*** | ***z*** | ***x*** | ***y*** | ***z*** |
| **Increasing** |  |  |  |  |  |  |  |  |  |
| Cuneus, middle occipital lingual gyrus  (BA 18, 19) | R/L | 193 | 3,77 | 22 | -80 | 2 | 22 | -77 | 6 |
|  |  |  | 3,68 | 30 | -72 | 2 | 30 | -70 | 5 |
|  |  |  | 3,55 | -10 | -92 | 6 | -10 | -89 | 10 |
| Thalamus | R/L | 17 | 3,37 | 6 | -4 | 2 | 6 | -4 | 2 |
|  |  |  | 3,32 | -2 | -8 | 2 | -2 | -8 | 2 |
|  |  |  | 3,19 | -2 | -16 | 10 | -2 | -15 | 10 |
| Superior frontal gyrus  (BA 6) | L | 27 | 3,35 | -22 | -8 | 66 | -22 | -8 | 66 |
|  |  |  |  |  |  |  |  |  |  |
| **Decreasing** |  |  |  |  |  |  |  |  |  |
| Cingulate gyrus, precuneus (BA 31, 23, 7) | R/L | 658 | 5,08 | -10 | -52 | 14 | -10 | -50 | 15 |
|  |  |  | 5,03 | -18 | -60 | 18 | -18 | -57 | 19 |
|  |  |  | 4,89 | 14 | -68 | 34 | 14 | -64 | 35 |
| Superior and middle temporal, supramarginal, angular, inferior parietal gyrus (BA 39, 40) | L | 303 | 4,95 | -46 | -60 | 26 | -46 | -57 | 27 |
|  |  |  | 4,94 | -58 | -32 | 42 | -57 | -29 | 40 |
|  |  |  | 4,70 | -42 | -52 | 34 | -42 | -49 | 34 |
| Sopramarginal, angular, inferior parietal gyrus  (BA 39, 40) | L | 198 | 4,02 | 62 | -48 | 34 | 61 | -45 | 34 |
|  |  |  | 3,98 | 54 | -52 | 30 | 53 | -49 | 30 |
|  |  |  | 3,70 | 62 | -32 | 42 | 61 | -29 | 40 |
| Inferior frontal gyrus  (BA 10) | R | 11 | 3,40 | 46 | 44 | -2 | 46 | 43 | -4 |

**Legend**: L= Left; R= Right; BA: Brodmann Area.
